# Assessment of real-world surveillance strategies for patients undergoing systemic therapy for brain metastases

**DOI:** 10.3389/fonc.2026.1815993

**Published:** 2026-04-20

**Authors:** Britney Weng, Caressa Hui, Jerica Lomax, Ahmed Mohyeldin, Nataliya Mar, Warren A. Chow, Misako Nagasaka, Jeremy Harris, Aaron B. Simon

**Affiliations:** 1 Department of Radiation Oncology, University of California Irvine, Irvine, CA, United States; 2 Division of Neuro-Oncology, University of California Irvine, Irvine, CA, United States; 3 Department of Neurosurgery, University of California Irvine, Irvine, CA, United States; 4 Division of Hematology and Oncology, University of California Irvine, Irvine, CA, United States

**Keywords:** brain metastases, CNS-penetrating systemic therapy, cranial brain radiation, radiosurgery, surveillance

## Abstract

**Introduction:**

Radiotherapy has long been first-line treatment for brain metastases. However, CNS penetrating systemic therapies are increasingly used as first-line alternatives. There is little evidence guiding surveillance in this setting. Here we retrospectively assessed surveillance strategies at a single institution and associated outcomes.

**Methods:**

Records from 33 patients, evaluated from 2021–2024 and treated with systemic therapy alone for active brain metastases, were reviewed. Time from diagnosis to 1st, 2nd, and 3rd surveillance MRI, incidence of intracranial progression, survival, and neurological adverse events were assessed. Patients were stratified by whether treatment was supported by the 2021 ASCO-SNO-ASTRO guidelines.

**Results:**

Treatment met guidelines for 14/33 patients. Median (range) time to 1st, 2nd, and 3rd MRI was 45 (18-207), 95 (46-204), and 180 (74-329) days, respectively. One-year cumulative incidence of local progression in the guideline group was 0.43 (95% CI [0.18-0.66]) and in the non-guideline group was 0.32 (95% CI [0.13-0.52], p=0.5). One-year cumulative incidence of brain radiation in the guideline group was 0.29 (95% CI [0.09-0.53]) and in the non-guideline group was 0.44 (95% CI [0.22-0.64], p=0.87). Two patients experienced safety events on systemic therapy (seizures).

**Discussion:**

Use of CNS-penetrating systemic therapies to treat brain metastases outpaced national guidelines in this study. While surveillance imaging frequency was variable and recurrences were common, many patients were able to delay radiation for over one year, with infrequent safety events. As interest in and options for CNS-penetrating systemic therapy grow, evidence-based guidelines for surveillance will be warranted.

## 1 Introduction

Brain metastases are a common manifestation of metastatic cancer. Due to their potential for morbidity and their privileged location behind the blood-brain barrier, local therapy has long played a central role in the management of brain metastases (1). Historically, whole brain radiation therapy (WBRT) was standard for most brain metastasis cases, with surgical resection reserved for select patients. More recently, stereotactic radiosurgery (SRS) has become standard for most patients due to its favorable cognitive toxicity profile (2, 3).

The recent development of targeted therapies and immunotherapies that penetrate the central nervous system (CNS) raises the possibility of controlling brain metastases without local therapy, potentially reducing treatment related costs and toxicities. Select CNS-penetrating therapies have been formally assessed in prospective clinical trials and demonstrated activity against brain metastases (4–8). As of the time of publication of the 2021 ASTRO-ASCO-SNO brain metastasis treatment guidelines, six drug-disease combinations were considered to have sufficient evidence of efficacy for appropriate use in the upfront management of brain metastases in well-selected patients (3). However, even for the best studied systemic agents, questions remain as to the conditions under which they should be used in place of local therapy (9).

In addition, new CNS-penetrating therapies are developed every year, outpacing the rate at which high quality brain metastasis-specific clinical trials can be performed and new guidelines can be developed. There is understandably considerable interest in the oncologic community in using new medications with activity in the CNS to treat brain metastases. However, there is little consensus about when to use them, how to follow patients undergoing treatment with them, and what to expect if patients progress on treatment.

To begin to understand how patients undergoing treatment with these medications are being followed in clinical practice, we looked retrospectively at patients from a single institution who deferred upfront radiation therapy for brain metastases to trial systemic therapy alone. Questions of interest included for which drug-disease combinations this strategy was employed, how frequently patients were monitored with magnetic resonance imaging (MRI), time-to and pattern of treatment failure, and whether patients experienced acute events due to tumor progression while deferring radiotherapy.

## 2 Methods

IRB approval was provided for this retrospective study. From the cohort of patients referred to the department of Radiation Oncology at our institution for brain metastases between 2021 and 2024, we identified patients who underwent systemic treatment alone as an initial strategy for new or progressive brain metastases. Because of the retrospective nature of the study, in some cases, patients had received prior radiation therapy to the brain, including whole brain radiation therapy or radiosurgery. In all cases the systemic treatments were being used to treat new or progressive brain lesions rather than stable or treated tumors. Patients who had received prior brain radiation therapy were not treated differently from those who had not in this analysis. In some cases, patients were referred to Radiation Oncology prior to initiation of the systemic therapy, while in others, patients were referred at later timepoints.

Patients were stratified by whether or not the systemic treatment strategy was supported by the 2021 ASCO-SNO-ASTRO treatment guidelines (3), which includes of the following: [1] osimertinib or icontinib for patients with EGFR-mutant non-small-cell lung cancer (NSCLC), [2] alectinib, brigatinib, or centrinib for patients with ALK-rearranged NSCLC, [3] pembrolizumab with pemetrexed and a platinum chemotherapy agent for patients with PD-L1 positive NSCLC, [4] Combination ipilimumab-nivolumab for patients with melanoma, [5] combination dabrafenib-trametinib for patients with BRAF-mutant melanoma, and [6] combination tucatinib-trastuzumab-capecitabine for patients with HER2-positive breast cancer.

For each patient in the cohort, baseline clinical information was collected, including primary cancer site, histology, driver mutation status, systemic therapies used, and number and size of brain metastases at time of systemic therapy initiation.

Key time-to event outcomes of interest for this study were the time from treatment initiation to first, second, and third surveillance MRIs. We also assessed the time to both local intracranial treatment failure (defined as the growth of any brain tumor that was active at the time of systemic treatment initiation) and distant intracranial treatment failure (defined as the finding of a new brain tumor, including leptomeningeal disease). These endpoints were assessed using a competing risk approach and Fine-Gray analysis, with discontinuation of the medication for reasons beyond intracranial progression (e.g. toxicity or extracranial progression) and non-CNS death or hospice enrollment defined as competing events. All time to event analyses use treatment initiation as time zero. Because of the retrospective nature of the study, determination of CNS progression was based on reported findings from the radiology reports, rather than on RECIST or RANO criteria. In rare cases where radiology reports were equivocal, documentation of the clinical determinations of the treating physicians at the time of the potential event was used for adjudication.

Additional outcomes of interest were [1] the rate at which CNS progression coincided with adverse events, defined as increased steroid use, surgical intervention, seizure, new neurological deficit, or neurologic death (defined as death caused by intracranial progression), [2] the next treatment strategy used for management of brain metastases after progression, [3] the pattern of intracranial progression, and [4] overall survival relative to patient specific predicted survival based on the calculated molecular graded prognostic assessment (GPA) and Eligibility Quotient (EQ) (10–13). Survival in the study cohort was compared with predicted survival using a previously described approach (14).

## 3 Results

### 3.1 Study cohort

Thirty-three patients were identified from the medical record. The characteristics of the study population are shown in **Table 1**. Median follow up was 507 days (range 70–2052 days). The drug-diagnosis combinations utilized are shown in **Supplementary Table 1**.

**Table 1:** Baseline characteristics of the patient cohort.

Characteristic	Guideline (n=14)	Non-guideline (n=19)
Primary site		
Breast	2 (14%)	7 (37%)
Lung	6 (43%)	7 (37%)
Melanoma	6 (43%)	2 (11%)
Kidney	0	2 (11%)
Rectum	0	1 (5%)
Sex		
Female	6 (43%)	10 (53%)
Male	8 (57%)	9 (47%)
Age (years)		
Median	55	55
Range	27-85	42-100
Graded prognostic assessment		
Median	2	2.5
Range	1-3	1-3.5
Eligibility quotient		
Median	0.43	0.55
Range	0.27-0.73	0.21-0.86
Number of brain metastases		
1	5 (36%)	9 (47%)
2-10	2 (14%)	8 (42%)
>10	7 (50%)	2 (11%)
Diameter of largest tumor (cm)		
Median	1.0	0.7
Range	0.5-2.6	0.4-3.4
Baseline Symptoms		
**Any symptoms at Baseline**	6 (43%)	2 (11%)
**Steroids Prescribed at Baseline**	7 (50%)	5 (26%)

Symptoms include headache, nausea, seizures, or focal neurological deficits attributed to brain metastases. Based on documented steroid prescriptions in electronic medical record at time of diagnosis.

### 3.2 Utilization of surveillance imaging

**Figure 1:**
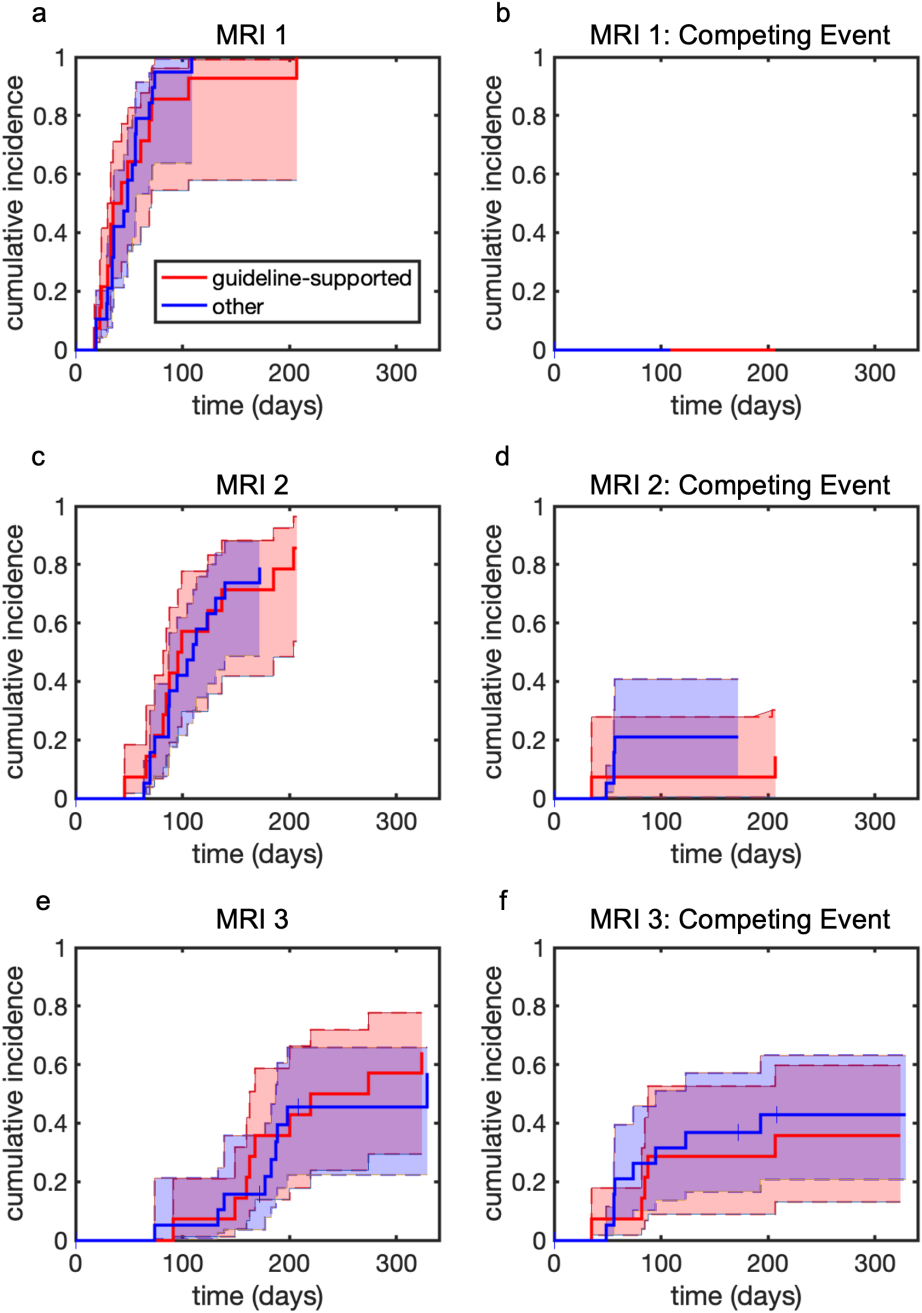
Time to first second, and third MRI vs. competing events. Shaded regions represent 95% confidence intervals. Tick marks represent censoring events. **(a)** Time to first MRI. **(b)** Time to competing event prior to first MRI. **(c)** Time to second MRI. **(d)** Time to competing event prior to second MRI. **(e)** Time to third MRI. **(f)** Time to competing event prior to third MRI.

All patients underwent at least one surveillance MRI after initiating systemic therapy. Median time to first surveillance MRI was 45 days (IQR 33–61 days, range 18–207 days). There was no significant difference in time to first MRI between the guideline-supported group and non-guideline supported group (p=0.83, **Figures 1a, b**). The cumulative incidence of completing at least two MRIs prior to progression or discontinuation was 0.82 (95% CI [0.64-0.82]), with an estimated 0.18 (95% CI [0.06-0.29]) experiencing a competing event prior to the second MRI. Of the patients that completed a second MRI, median time to second MRI was 95 days (IQR 78–124 days, range 46–204 days). There was no significant difference in time to second MRI between the guideline supported group and non-guideline supported group (p=0.92, **Figures 1c, d**). The cumulative incidence of completing at least three MRIs was 0.60 (95% CI [0.38-0.71]), with an estimated 0.40 (95% CI [0.23-0.56]) experiencing a competing event prior to the third MRI. Of the patients that completed a third MRI, median time to third MRI was 180 days (IQR 152–200 days, range 74–329 days). There was no significant difference in time to third MRI between the guideline supported group and non-guideline supported group (p=0.56, **Figures 1e, f**).

### 3.3 Pattern of treatment failure

**Figure 2:**
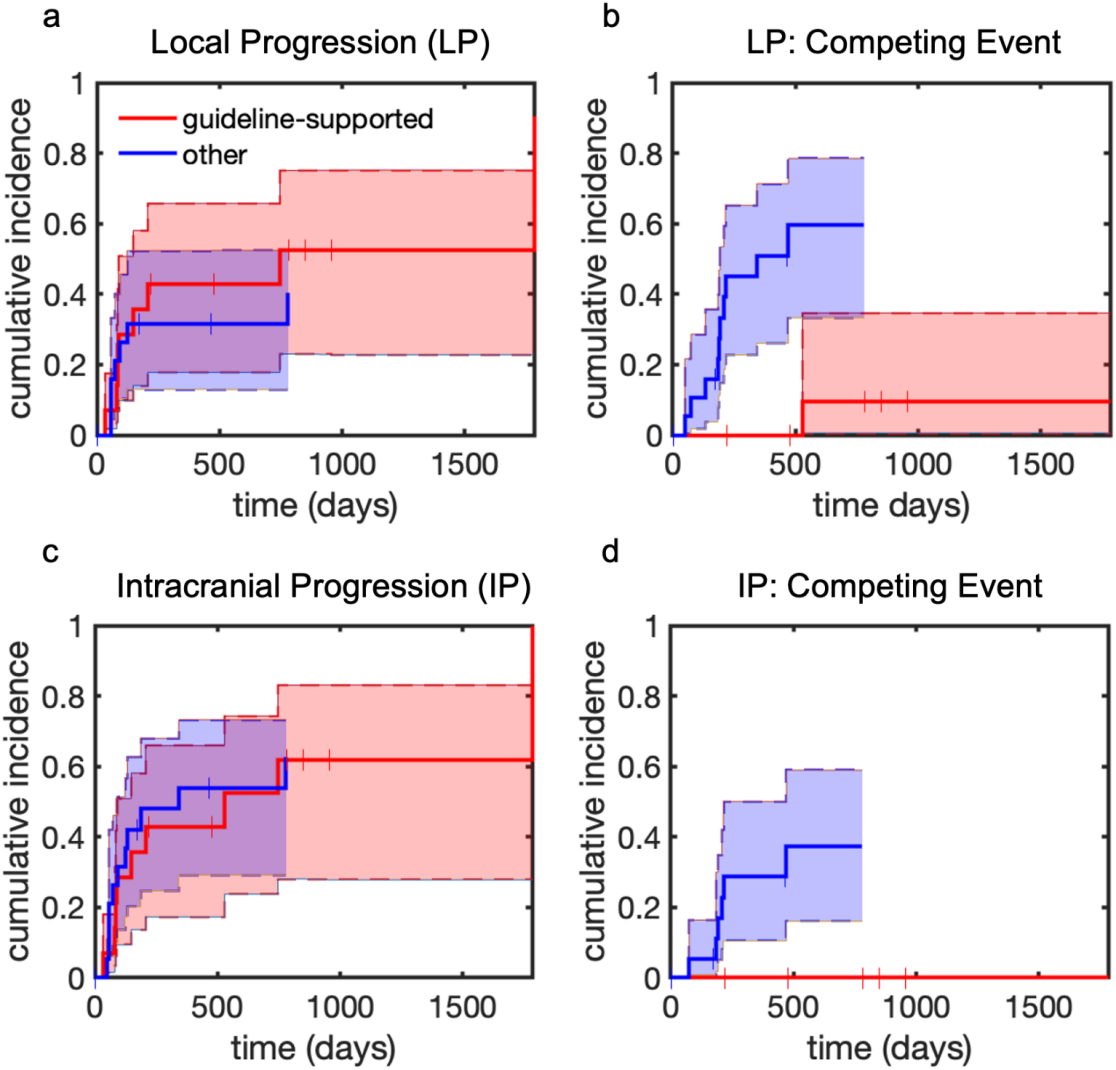
Cumulative incidence of intracranial local progression (LP) and any intracranial progression (IP). Shaded regions represent 95% confidence intervals. Tick marks represent censoring events. **(a)** local progression. **(b)** competing events prior to local progression. **(c)** Intracranial progression. **(d)** competing events prior to intracranial progression.

There was no significant difference in the cumulative incidence of local brain progression as site of first treatment failure between the guideline-supported and non-guideline-supported cohorts (p=0.59, **Figure 2a**). However, there was a statistically significant difference in the cumulative incidence of competing events (p=0.003, **Figure 2b**). The one-year cumulative incidence of local brain progression in the guideline supported group was 0.43 (95% CI [0.18-0.66]) and in the non-guideline-supported group was 0.32 (95% CI [0.13-0.52]). There were no competing events (including distant brain progression) at one year in the guideline-supported group. In the non-guideline-supported group, the cumulative incidence of competing events at one year was 0.51 (95% CI [0.27-0.71]).

There was no significant difference in the rate of any brain progression (local or distant) as a site of first treatment failure between the guideline-supported and non-guideline-supported cohorts (p=0.79, **Figure 2c**). However, there was a statistically significant difference in the rate of competing events (p=0.016, **Figure 2d**). In the guideline-supported group, all brain progression within the first year included local progression. Thus, the one-year cumulative incidence of any brain progression in the guideline supported group was 0.43 (95% CI [0.18-0.66]). In the non-guideline-supported group the cumulative incidence was 0.54 (95% CI [0.29-0.73]). There were no competing events at one year in the guideline-supported group. In the non-guideline-supported group, the cumulative incidence of competing events at one year was 0.29 (95% CI [0.1-0.50]).

### 3.4 Management strategies following progression or discontinuation of systemic therapy

At the time of last follow up, 7/33 (21%) of patients remained on the initial systemic therapy (**Table 2**). Six patients (18%) discontinued systemic therapy due to side effects. The most common subsequent treatment strategy was radiation therapy, with stereotactic radiosurgery employed in 11/33 patients (33%) and whole brain radiation used in 2/33 patients (6%). New systemic therapy or increased systemic therapy dose was used in 8/33 patients (24%), and 5/33 patients (15%) either opted for supportive care alone after progression or died while on treatment.

**Table 2:** Treatment strategies following progression or discontinuation of systemic therapy.

Treatment strategy	Guideline (n=14)	Non-guideline (n=19)
**No Progression/Discontinuation**	**5 (36%)**	**2 (11%)**
**Progression/Discontinuation**	**9 (64%)**	**16 (84%)**
New systemic therapy	2 (14%)	5 (26%)
Increased Dose	1 (7%)	0
Stereotactic Radiosurgery	5 (36%)	6 (32%)
WBRT/HA-WBRT	1 (7%)	1 (5%)
Supportive Care	0	4 (21%)
**Death On Treatment**	**0**	**1 (5%)**

Patients continued on systemic therapy without evidence of progression at last follow up.

Patients either experienced a documented progression event or discontinued systemic therapy for toxicity.

Patients died without evidence of progression or discontinuation of systemic therapy.

**Figure 3:**
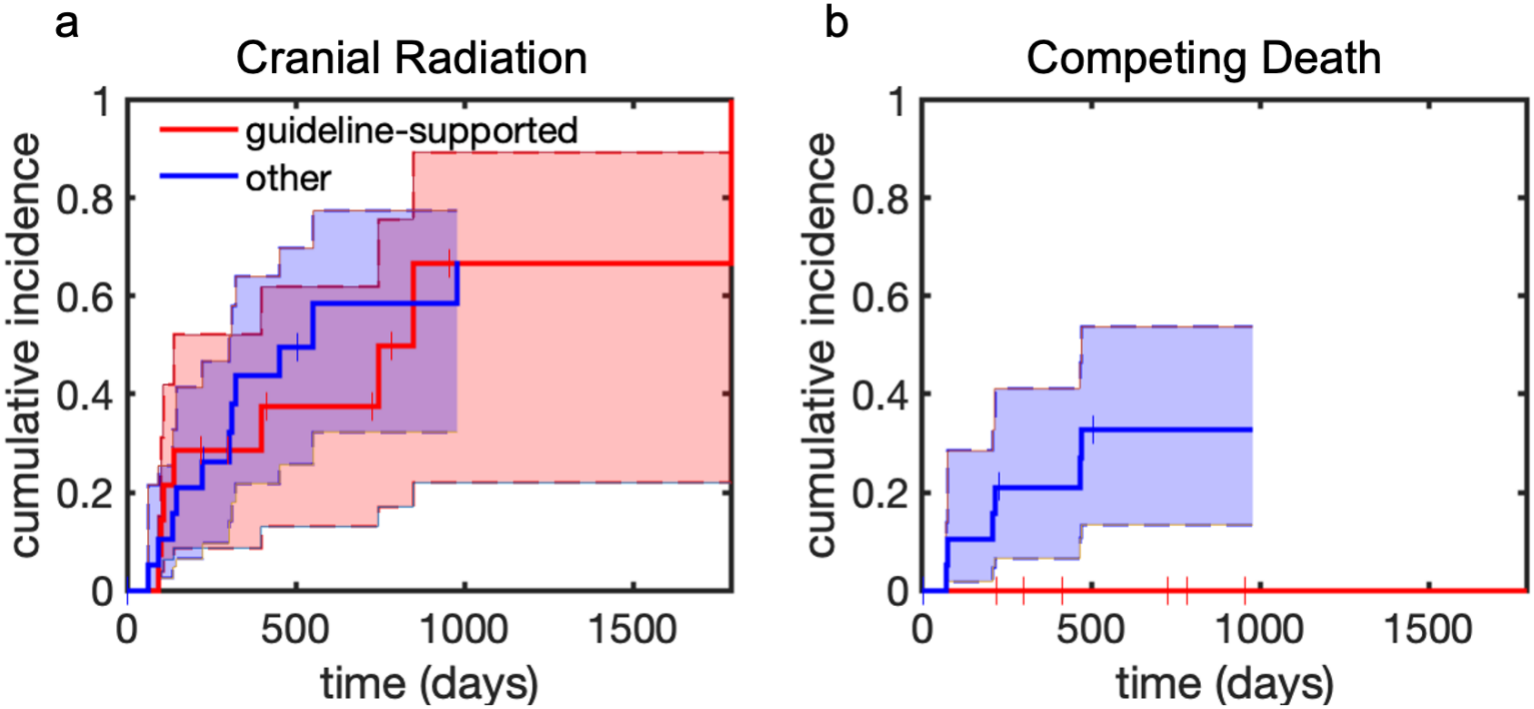
Cumulative incidence of brain radiation after initiation of systemic therapy. Shaded regions represent 95% confidence intervals. Tick marks represent censoring events. **(a)** Cumulative incidence of cranial radiation. **(b)** Competing death prior to cranial radiation.

There was no significant difference in time to undergoing brain radiation therapy between the guideline-supported and non-guideline-supported cohorts (p=0.87, **Figure 3a**). However, there was a statistically significant difference in the cumulative incidence of competing death (p=0.03, **Figure 3b**). The one-year cumulative incidence of brain radiation in the guideline supported group was 0.29 (95% CI [0.09-0.53]) and in the non-guideline-supported group was 0.44 (95% CI [0.22-0.64]). There were no death events prior to brain radiation in the guideline-supported group. In the non-guideline-supported group, the cumulative incidence of competing death at one year was 0.21 (95% CI [0.07-0.40]). One patient with melanoma who had a persistent but stable lesion underwent consolidation radiosurgery 849 days after diagnosis. All other patients underwent radiation only after intracranial progression.

### 3.5 Adverse events

One death occurred while a patient was undergoing systemic therapy. However, the death was attributed to aspiration in the setting of hemoptysis, without any prior evidence of intracranial progression. Two cases of seizure occurred while patients were undergoing systemic therapy. In one case, a post-seizure MRI showed no evidence of intracranial progression, and the patient continued systemic therapy without further incidence of seizures. In the second case, a patient with innumerable metastases, who underwent initial treatment with combination BRAF-MEK-inhibitors for BRAF-mutant melanoma, transitioned to ipilimumab-nivolumab and experienced a seizure one month after discontinuing the BRAF-MEK-inhibitors. The patient was hospitalized and started on anti-epileptic therapy and steroids. Imaging showed progression of intracranial disease. The patient restarted the combination BRAF-MEK-inhibitors and subsequent imaging showed treatment response. No other patients required new brain-related medical therapy at time of progression. No patients required surgical intervention at the time of progression.

### 3.6 Overall survival

**Figure 4:**
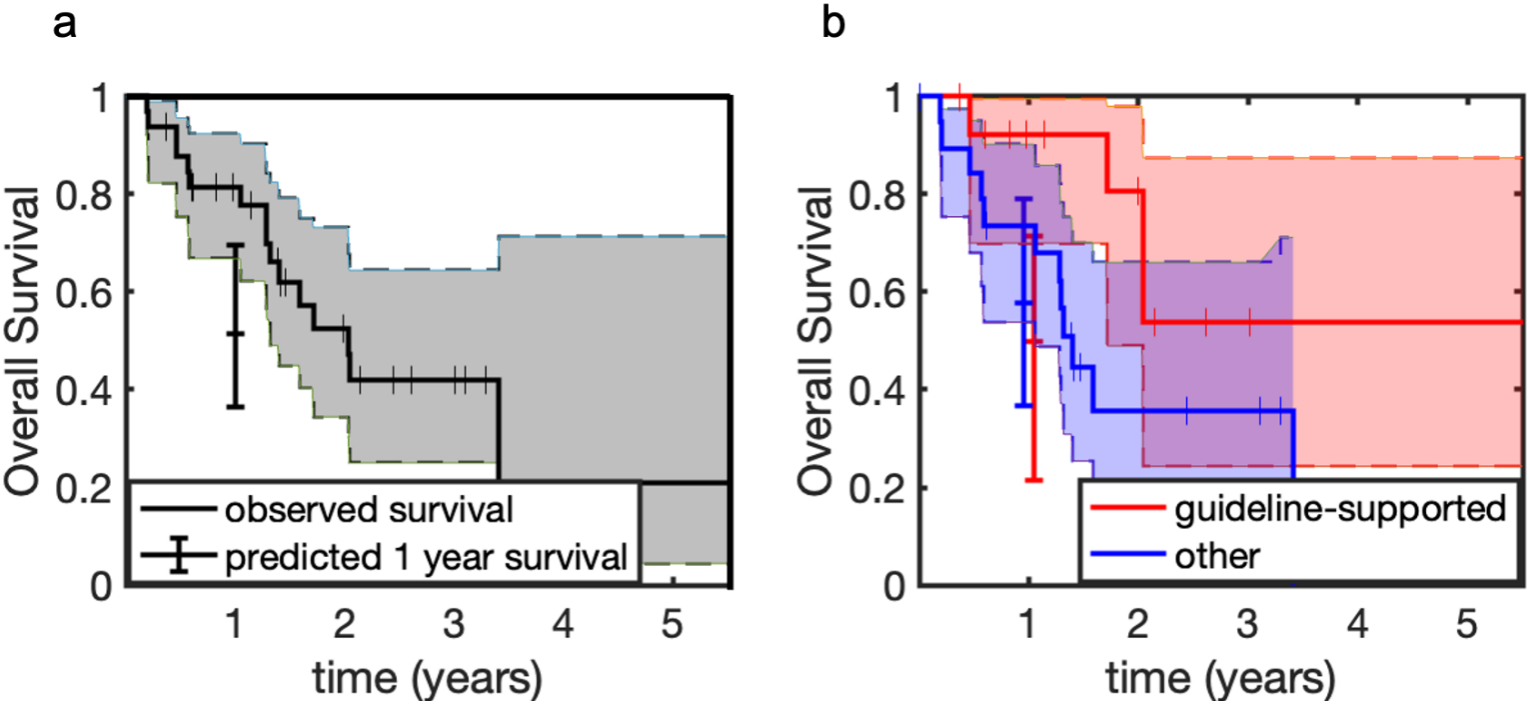
Observed vs predicted one-year overall survival for **(a)** the whole cohort and **(b)** the cohort stratified by treatment. Shaded regions represent 95% confidence intervals. Tick marks represent censoring events.

Median survival from the time of start of active surveillance was 24.5 months for the whole cohort (95% CI 15.8 months – not reached) (**Figure 4a**). The observed 1-year survival for the whole cohort was 0.81 (95% CI 0.67-0.92). This was significantly greater than the 1-year predicted survival based on molecular graded prognostic index and eligibility quotient, which was 0.52 (95% CI 0.36-0.69). There was a trend towards longer overall survival in the guideline supported group than the non-guideline supported group, with univariate Cox Proportional Hazard Ratio of 0.35 (p=0.07) (**Figure 4b**). The observed 1-year survival for the guideline supported group was 0.92 (95% CI 0.71-1.0). The observed 1-year survival for the non-guideline supported group was 0.74 (95% CI 0.53-0.90).

## 4 Discussion

The management of brain metastases has changed significantly in the last 25 years. Since the publication of RTOG 90-05 (15), stereotactic radiosurgery has replaced whole brain radiation therapy as the standard first-line treatment for most patients with brain metastases. Importantly, radiosurgery was adopted not because it improved intracranial disease control, but because it improved patient cognitive outcomes (16–18). Further, the clinical use of radiosurgery to treat patients with >4 brain lesions long preceded the publication of randomized trials supporting that strategy (19–21).

The clinical adoption of novel systemic therapies with activity against brain metastases may take a similar course. Since the approval of Osimertinib for patients with metastatic EGFR-mutated NSCLC (22), numerus drugs with activity in the CNS have received approval for treatment of metastatic cancers, with regulatory approval outpacing the completion of brain metastasis-specific trials. The appeal of using these medications upfront for brain metastases is clear. Delaying initiation of radiotherapy has the potential to reduce treatment costs and treatment toxicity. While it remains critical that prospective trials be conducted to determine which agents are most effective in the CNS, simultaneously developing evidence-based guidelines for the surveillance of patients deferring brain radiation therapy may increase patient safety and improve outcomes.

As a first step in this process, we retrospectively assessed the surveillance protocols used to manage patients at a single institution who underwent systemic therapy alone for active brain metastases, as well as the patients’ clinical outcomes. We found multiple of CNS-penetrating therapies were being used to treat brain metastases across a broad set of cancer histologies, suggesting that the clinical implementation of radiotherapy deferral strategies is outpacing the publication of national guidelines. We found a wide variance in the frequency of surveillance imaging in this cohort, with a median time to first MRI of approximately 6 weeks, but a range from just over two weeks to over 6 months. Similarly, there was considerable heterogeneity in follow up imaging schedules. While the median patient completed three MRIs in approximately six months, similar to the NCCN recommendations for post-SRS surveillance (2), some patients underwent three MRIs within less than three months, while others took nearly a year to complete three scans. While some of this heterogeneity likely reflects challenges that occur in real-world practice (e.g. insurance delays, loss to follow up, co-morbid illness), it also suggests that there is a lack of consensus regarding how frequently surveillance imaging should be conducted for patients undergoing systemic treatments for brain metastases. Interestingly, there was not a significant difference in the imaging strategies used to manage patients who were undergoing treatment with guideline-supported treatment regimens vs. other regimens, suggesting that clinicians may not be differentially surveilling patients based on recognition status in national guidelines.

Despite the heterogeneity in surveillance, we did not an observe an overt signal that deferring upfront radiotherapy in favor of a systemic therapy trial was unsafe, at least in the short term. Intracranial progression was a frequent event in this cohort, with 43% of the guideline-supported group and 32% of the non-guideline supported group experiencing local brain progression within one year. However, despite the frequency of intracranial progression events, only two CNS-related significant adverse events were observed, both of which were seizures, and only one of which was associated with imaging evidence of intracranial progression. This frequency is comparable to the rate of post-radiosurgery seizures reported in the literature (23).

While the retrospective nature of this study makes a comparative analysis of overall survival outcomes in this cohort inherently challenging, we sought to determine if we could detect a signal that patient outcomes in this group were significantly worse than what might be expected of similar patients treated with other approaches. To accomplish this, we compared the rate of survival at one year to each patient’s expected rate of one-year survival based on their molecular GPA. Reassuringly, we did not find that observed overall survival was lower than expected survival. Instead, survival in this cohort was significantly greater than predicted. This does not imply that deferring radiation therapy confers a survival benefit, as this cohort may differ from the cohort used to develop the molecular GPA, leading to selection bias that is not captured by GPA. However, the molecular GPA remains a widely used and accepted prognostic tool for patients with brain metastases, and, as such, this finding is encouraging.

It is important to emphasize that stereotactic radiosurgery remains the standard of care front-line treatment for most patients with limited brain metastases, including many patients who are eligible for CNS-penetrating systemic therapies. WBRT and surgical resection also continue to have important roles in the upfront treatment of appropriately selected patients (2). Local control remains an important endpoint in brain metastasis management, and, while randomized trials are lacking, well-designed retrospective studies continue to demonstrate local-control benefits to treatment with upfront SRS for patients undergoing CNS-penetrating targeted therapies (9). Further, there continues to be active research into the potential for upfront SRS to act synergistically with immunotherapy agents to prime the immune system in an antineoplastic fashion (24). For patients that do defer local treatments such as SRS to trial systemic therapies, it remains critical that they undergo standard treatments promptly at the time of progression.

There are multiple limitations to this study. First, it is a small, retrospective study conducted at a single institution, which render the findings hypothesis-generating, rather than definitive and may limit their generalizability. The multiple endpoints examined may also lead to an underestimation of the associated type-1 error. Second, the method by which patients were identified for inclusion, based on their referral to radiation oncology, may skew the composition of the cohort. It is possible that additional unidentified patients are undergoing systemic therapy treatment at this institution for brain metastases without referral to radiation oncology. However, because hospitalization for brain metastases dependably triggers a radiation oncology consultation at this institution, it is likely that the study cohort is biased towards patients who are more likely to experience adverse events, which is reassuring. Third, as discussed above, the retrospective nature of the study precludes rigorous comparative analysis against cohorts that underwent upfront radiation therapy. Fourth, the decision to stratify patients based on the 2021 ASCO-SNO-ASTRO Guidelines was pragmatic, based on its publication at the time the study period began, its endorsement by multiple national professional societies, and its explicit listing of drug-disease combinations. It is recognized that over the study period, additional evidence in support of other systemic therapies has accumulated, and that an updated guideline might include therapies that were considered “non-guideline-supported” for the purposes of this study. However, in an effort to be as objective as possible, no attempt was made by the authors to add additional treatment options to the “guideline-supported” group. The decision to stratify patients in this manner should thus not be taken as an endorsement by the authors of using these guideline-supported therapies vs. non-guideline supported therapies for the treatment of brain metastases. Fifth, determination of local progression relied on the imaging reports written and clinical determinations made at the time of image acquisition. While such an approach is not as reproducible as standardized methods such as RECIST or RANO, it is practical and pragmatic for a study in which clinical decisions, such as the decision to treat a patient with radiation or change systemic therapy, were made based on those determinations. As the goal of this study was to assess how these medications were used in a real clinical setting, this approach is consistent with the intent of this study. Sixth, while efforts were made to comprehensively derive adverse events from the medical record, due to the retrospective nature of the study, events may still be under reported. Finally, while the study demonstrated that a majority of patients in the cohort were able to defer radiation therapy for over one year, it was not possible to assess whether delaying treatment in this way had a beneficial effect on either cognitive function or quality of life. A prospective, randomized trial, with rigorous quality of life assessment and cognitive function testing, will be required to demonstrate any benefit in these domains.

## 5 Conclusion

To our knowledge, this is the first study to assess active surveillance as a generalized strategy for brain metastasis management, rather than as a strategy for a particular drug-class-disease combination. We found that CNS-penetrating therapies were widely implemented as treatments for active brain metastases, with indications outpacing national guidelines. We found that surveillance imaging frequency in this setting was highly variable, suggesting the need for evidence-based surveillance strategies that can keep pace with this rapidly developing field. We found that local recurrences were common, indicating a need for close surveillance. However, we did not observe a high frequency of safety events or an early-death signal. Together, these findings generate the hypothesis that CNS-penetrating systemic therapies, even when only modestly effective, have the potential to be utilized safely with the goal of delaying brain radiation. A prospective, randomized trial of active surveillance vs. upfront radiation therapy, in a drug and histology agnostic study population, may help to determine whether radiotherapy delay improves cognitive function, quality of life, or costs of care and to verify that it has are no detrimental effects on survival.

## Data Availability

The raw data supporting the conclusions of this article will be made available by the authors, without undue reservation.
