# Supplementary material for: Assessment of real-world surveillance strategies for patients undergoing systemic therapy for brain metastases
Source: Front Oncol. 2026 Apr 20;16:1815993. doi: 10.3389/fonc.2026.1815993 (PMC13135992; doi:10.3389/fonc.2026.1815993)
Supplement: Supplementary file 1 [file DataSheet1.pdf]

Supplementary Table 1: Systemic therapies used in the study cohort

Systemic Therapy Agent	Primary Site	Under ASCO-SNO-ASTRO Guidelines	Patients
Abemaciclib and fulvestrant	Breast	No	1
Afatinib	Lung	No	1
Amivantamab and lazertinib	Lung	No	1
Axitinib	Kidney	No	2
Capecitabine	Breast	No	1
Capecitabine, tucatinib, and trastuzumab	Breast	Yes	2
Capmatinib	Lung	No	1
Dabrafenib and trametinib	Melanoma	Yes	1
Encorafenib and binimetinib	Melanoma	Yes	1
Ipilimumab-nivolumab	Melanoma	Yes	4
Lorlatinib	Lung	No	1
Osimertinib	Lung	Yes	6
Pembrolizumab	Melanoma	No	3
Sirolimus	Lung	No	1
Trastuzumab	Breast	No	2
Trastuzumab and fulvestrant	Breast	No	1
Clinical trial drug regimen	Breast	No	2
	Lung	No	1
	Rectum	No	1
